# Prenatal Prediction of Outcome by Fetal Gastroschisis in a Tertiary Referral Center

**DOI:** 10.3390/diagnostics10080540

**Published:** 2020-07-30

**Authors:** Katharina Nitzsche, Guido Fitze, Mario Rüdiger, Cahit Birdir

**Affiliations:** 1 Department of Obstetrics and Gynecology, University Clinic of Carl Gustav Carus Dresden, Technische Universität Dresden, 01307 Dresden, Germany; katharina.nitzsche@ukdd.de; 2 Department of Pediatric Surgery, University Clinic of Carl Gustav Carus Dresden, Technische Universität Dresden, 01307 Dresden, Germany; guido.fitze@ukdd.de; 3 Department of Pediatrics, University Clinic of Carl Gustav Carus Dresden, Technische Universität Dresden, 01307 Dresden, Germany; mario.ruediger@ukdd.de

**Keywords:** gastroschisis, exomphalos, omphalocele, prenatal diagnosis, surgery

## Abstract

The aim of this study was to find a prenatal parameter to be able to predict possible prenatal complications or postnatal surgical options, thus allowing the fetal medicine specialist, together with pediatric surgeons and neonatologists, to improve the counseling of the parents and to determine the timing of delivery and therapy. This was a retrospective analysis of prenatal diagnosis and outcome of fetuses with 34 cases of gastroschisis between the years 2007 and 2017. A total of 34 fetuses with gastroschisis were examined and 33 outcomes registered: 22 cases of simple gastroschisis (66.7%) and 11 cases of complex gastroschisis (33.3%). A cut-off value of 18 mm for intraabdominal bowel dilatation (IABD) showed a positive predictive value (PPV) of 100% for predicting simple gastroschisis. IABD gives the best prediction for simple versus complex gastroschisis (cut-off of 18 mm). Extra-abdominal bowel dilatation (EABD) cut-off values of 10 mm and 18 mm showed low sensitivity and specificity to predict complex gastroschisis.

## 1. Introduction

One of the most commonly seen abdominal wall defects of the newborn is gastroschisis. Gastroschisis is a right-sided paraumbilical abdominal wall defect which causes the herniation of the bowel, which floats in amniotic fluid. The lateral abdominal walls probably fail to fuse at the embryological period until 5 weeks into pregnancy [1]. The prevalence of gastroschisis is 3.8 to 4.5 per 10,000 births [2,3], but there has been an increase in the number of cases in the last few decades [4]. The associated complications are mostly in the gastrointestinal area, seen as intestinal obstruction or atresia [5,6], and are defined as complex gastroschisis compared to simple gastroschisis without complications. Chromosomal abnormalities or co-morbidities are seen rarely [7]. A multidisciplinary collaboration, with regular meetings between fetal medicine specialists, obstetricians, neonatologists and pediatric surgeons, is needed for the diagnosis, counseling the parents, ultrasound controls and decision making about the time of delivery.

The aim of this study was to find a prenatal parameter to be able to predict possible prenatal complications or postnatal surgical options, thus allowing the fetal medicine specialist, together with pediatric surgeons and neonatologists, to improve the counseling of the parents and determine the timing of delivery and therapy.

## 2. Materials and Methods

The pregnancies complicated with gastroschisis were examined retrospectively for evaluation of the diagnosis, outcome of the pregnancy and genetic diagnosis between 2007 and 2017 at our tertiary center. All pregnancies were examined with two Voluson E8 machines (General Electric) or Epiq 7 (Philips, Amsterdam, Holland) by consultants of fetal medicine who are certified by the Fetal Medicine Foundation (FMF) of London or the German Association for Ultrasound in Medicine (DEGUM Level II). An anomaly scan was performed for all patients to exclude other associated anomalies. After diagnosing the cases, an invasive genetic diagnostics was offered and performed when approved.

The cases included in this study were diagnosed in our department. After the diagnosis, perinatal counseling was offered to the families together with fetal medicine specialists, obstetricians, pediatricians and pediatric surgeons. All the babies were born at our department, and neonatal care was given at the Department of Neonatology in our hospital. The surgical management was performed at the Department of Pediatric Surgery in our hospital. The digital documentation relating to the scans, delivery, operations and patient folders was examined.

**Figure 1:**
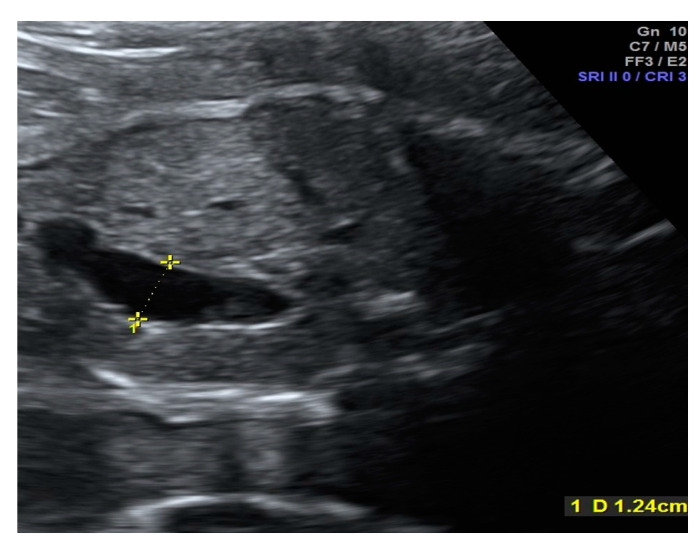
Gastroschisis: example of intraabdominal bowel dilatation (IABD).

**Figure 2:**
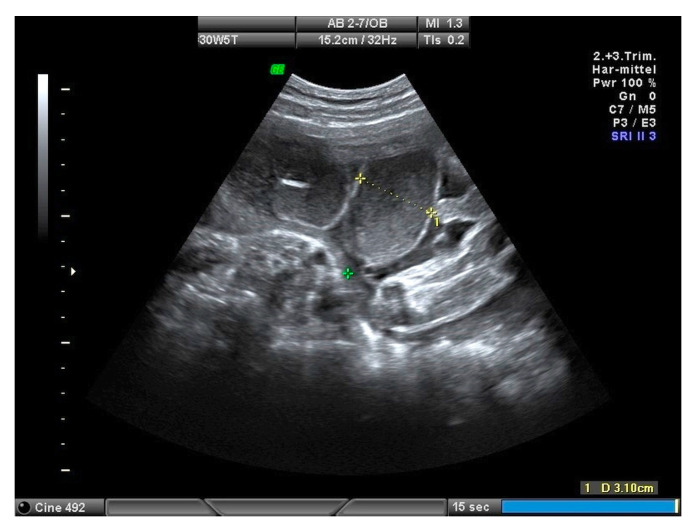
Gastroschisis: example of extra-abdominal bowel dilatation (EABD).

The fetuses with gastroschisis were divided into two groups, simple or complex gastroschisis (gastroschisis combined with intestinal stenosis, atresia or perforation), by analyzing the data from the prenatal scans, surgical reports of the pediatric surgeons and postnatal outcome. The ultrasound scans were performed every four weeks until 30 weeks of pregnancy. After 30 weeks of pregnancy, ultrasound scans were performed weekly until delivery. The fetal growth, Doppler measurements, assessment of the amniotic fluid using the deepest pocket, the maximal intraabdominal bowel dilatation (IABD) and extra-abdominal bowel dilatation (EABD) were assessed during the scans. Using the saved images of the last scan between 30 to 34 weeks of pregnancy retrospectively, the same examiner performed the measurements of IABD and EABD. Cut-offs of 10 mm for IABD and 18 mm for EABD were used, as previously published by Langer et al. [8] and Carnaghan et al. [9], to predict complex gastroschisis. IABD and EABD were measured using the short axis. The most dilated bowel segment was measured. Inner-to-inner wall measurement was performed (Figure 1 and Figure 2). Polyhydramnios was defined when the deepest single pocket measurement was above 8 cm [10].

Furthermore, the clinical outcome was evaluated for all the cases with gastroschisis. The sensitivity, specificity and positive predictive value (PPV) of the above-mentioned cut-off values were assessed. For the statistical analysis, Excel was used (Microsoft Corporation, Redmond, WA, USA, 2010). The ethics committee of the Technische Universität Dresden, Germany approved this study (BO-EK-66022020, 11 March 2020).

## 3. Results

Between 2007 and 2017, in the Department of Obstetrics and Gynecology at our tertiary center, 23,934 women delivered 25,107 babies.

Fetal gastroschisis was diagnosed in 34 cases and the fetal outcome was obtained in 33 cases with 33 live births, with a prevalence of 1.3/10,000 in our collective (Table 1). One patient delivered elsewhere. The median maternal age was 23 (between 17 and 37). The first examination in our hospital was carried out between 11+4 and 31+0 weeks of pregnancy. After fetal diagnosis, 12 patients asked for amniocentesis (35.3%) and all pre- and postnatal karyotyping showed a normal karyotype. The fetal anomaly scan showed urogenital anomalies in two cases (6.1%): one fetus with agenesis of one kidney and one fetus with ureteropelvic obstruction. In these two cases, the fetal karyotyping was not performed. In all 33 pregnancies, a therapy with corticosteroids was given to prevent infant respiratory distress syndrome since all the fetuses were delivered between 33+0 and 34+5 weeks of pregnancy via C-section. The average birth weight was 2190 g (1370–2985 g) at around the 40th percentile (20%–90%). Primary closure of the defect was possible in all cases on the day of delivery. There were 22 out of 34 babies (66.7%) with simple gastroschisis and 11 with complex gastroschisis (33.3%).

The previously published sonographic cut-off values (using the last scan after 30 weeks of pregnancy) of 10 mm for IABD and 18 mm for EABD were used in our collective respectively to predict complex gastroschisis postnatally [9,11]. An IABD measurement below 10 mm showed simple gastroschisis in 20 babies (90.9%) and complex gastroschisis in eight babies (72.7%) (Figure 1). IABD ≥ 10 showed simple gastroschisis in two babies (9.1%) and complex gastroschisis in three babies (27.3%). This prediction model for complex gastroschisis had a sensitivity of 27.3% and specificity of 90.9%. IABD cut-offs <18 mm and ≥18 mm showed a sensitivity of 27.3% and a specificity of 100% since none of the babies had simple gastroschisis with an IABD ≥18 mm.

An EABD below 10 mm showed simple gastroschisis in seven babies (31.8%) and complex gastroschisis in three babies (27.3%). EABD ≥ 10 mm showed simple gastroschisis in 15 babies (68.2%) and a complex gastroschisis in eight babies (72.7%). The sensitivity and specificity to predict complex gastroschisis was 72.7% and 31.8%, respectively. EABD cut-offs below and above 18 mm showed a sensitivity and specificity of 45.5% and 72.7%, respectively (Table 2). Two fetuses with complex gastroschisis developed polyhydramnios in pregnancy, whereas none of the fetuses had polyhydramnios with simple gastroschisis. The analysis of saved ultrasound images showed increasing intestinal dilatation up to an EABD > 30 mm in one of the cases with complex gastroschisis and the follow-up scans showed a decrease in EABD after a perforation (Figure 2).

A comparison of the outcomes by gastroschisis can be seen in Table 1 and Table 2.

## 4. Discussion

Gastroschisis is one of the most commonly seen abdominal wall defects of the fetus and newborn. Gastroschisis has an incidence in 3.7–4.5/10,000 births [2,3]. Our collective showed a prevalence of 13/10,000 for gastroschisis. This prevalence is higher than previous studies, probably due to the nature of the clinic, which is a tertiary reference center with a fetal medicine unit.

The postnatal outcome of newborns with gastroschisis depends on the prenatal damage of the intestines and damage-related postnatal function [9,11,12]. This defect is also correlated with younger pregnant women. Lausman et al. [13] found a significant younger population of pregnant women (below 21 years of age) with fetuses with gastroschisis (42% versus 7.3%) and Mastroiacovo et al. [7] found a median age of 21.9.

Chromosomal abnormalities are seen rarely in gastroschisis; therefore, there is no genetic testing recommended [14]. Mastroiacovo et al. reported 41 cases out of 469 (8.7%) with chromosomal abnormalities when there was another anomaly seen and gastroschisis was not isolated. Genetic testing should be carried out in these cases [7]. We could not diagnose any chromosomal aberrations in our collective, but in two cases with combined anomalies, the karyotyping was not accepted to be performed by the pregnant women. The most common combined anomaly seen is the anomaly of the gastrointestinal tract as intestinal atresia, stenosis and perforation, which is defined as complex gastroschisis [9]. The prevalence of complex gastroschisis is given as between 12.8% and 14.9% in the literature [5,9,11,15]. In our collective, 11 out of 34 cases (33.3%) had complex gastroschisis. Fetuses with gastroschisis present fetal growth retardation frequently and are reported as small for gestational age (SGA) fetuses with normal Doppler parameters [16,17]. We could not confirm this result in our collective.

The prevalence of intrauterine fetal death (IUFT) is given as 4.48/100 pregnancies and 1.28/100 after 36 weeks of pregnancy in a meta-analysis published by South et al. [18]. The highest risk of IUFT is seen before 36 weeks of pregnancy and the prevalence does not increase after 35 weeks. We did not observe any IUFT in our collective. The rate of premature delivery was given as 28% by Lausman et al. [13] and the mean pregnancy week for delivery was 36+0 weeks according to Kuleva et al. [19].

All pregnant women were delivered via C-section as premature deliveries in our collective to prevent intrauterine inflammation of the intestines and the damage caused by a vaginal delivery and to enable primary closure of the defect postnatally. The time and mode of delivery are discussed heterogeneously in the literature. The reason for the difference is driven by the assumption that an advanced week of pregnancy triggers inflammation of the intestinal wall due to inflammatory mediators in amniotic fluid affecting the postnatal therapy and outcome of the newborns [20,21,22,23]. Serra et al. favors an elective C-section after 34 weeks of pregnancy and after administering therapy with corticosteroids to prevent infant respiratory distress syndrome [24]. We prefer this approach in our clinic, together with the Department of Pediatric Surgery, due to a better outcome with reduced inflammation of the intestines and thus better conditions for a primary closure.

The other studies did not report this advantage of premature delivery [8,17,25]. Premature delivery is also related to the C-section [24,25]. The advantages of a C-section versus vaginal delivery are discussed controversially, and the advantages as well [24,26] as disadvantages of a C-section for the outcome of the newborn were reported [27,28].

The prenatal prediction of a complex gastroschisis is hard but important due to the increased rate of complications compared to simple gastroschisis [6,29]. Many research groups measured the intra- and extra-abdominal dilatation of the intestines due to the predictive value of increase in dilatation with increasing stage of pregnancy to predict the postnatal function of the gastrointestinal tract [9,11,12,30].

The cut-off value of 18 mm for IABD showed a sensitivity of 27.3% and PPV of 100% (when IABD was above 18 mm) to predict complex gastroschisis and a specificity of 100% to predict simple gastroschisis in our collective compared to the studies of Carnaghan et al. (PPV 25%) [9] and Ghionzoli et al. (PPV 22%) [11]. The higher PPV of IABD in our cohort is probably due to the higher prevalence of complex gastroschisis in this series as compared to other studies.

The cut-off values of EABD were not suitable for predicting complex gastroschisis in our collective since an EABD value ≥18 mm (PPV of 50% to predict complex gastroschisis) could only predict in 45.5% of fetuses with complex gastroschisis and 27.3% of fetuses with simple gastroschisis. These values were parallel to the study of Carnaghan et al. (PPV 24%) [9].

In three cases, both IABD and EABD were ≥18 mm, and in all cases, there was complex gastroschisis with intestinal stenosis. In one of the cases, we observed polyhydramnios, and an intestinal perforation was observed postnatally. Carnaghan et al. [9] reported a PPV of 75% for complex gastroschisis when both IABD and EABD were measured. Ghionzoli et al. [11] reported significantly more complex cases of gastroschisis when polyhydramnios was seen. We could exclude complex gastroschisis in our collective when the fetal intra-abdominal intestinal dilatation was smaller than 18 mm. We did not measure the performance of prediction in a combined approach using polyhydramnios since we had only two cases with polyhydramnios.

The combination of different ultrasound parameters like IABD, EABD, amniotic fluid levels and serial measurements of intestinal dilatation could be important to predict simple or complex gastroschisis in planning the optimal perinatal approach. The combined approach to predict simple or complex gastroschisis did not improve the predictive value in our cohort, probably due to the small number of cases (Table 2).

Prospective studies are needed in order to evaluate the diagnostic accuracy and reproducibility of IABD and EABD assessment in predicting postnatal outcomes of fetuses prenatally detected with gastroschisis. A recent large, prospective, longitudinal study showed that increased IABD measured on at least three occasions can differentiate between simple and complex gastroschisis, but the PPV was low, and therefore the clinical usefulness of this marker is limited [31]. Nevertheless, Andrade et al. reported that measurement of IABD at 20–22 or at 30–32 weeks’ gestation was useful in the prediction of complex gastroschisis [32].

The limitation of this study was the retrospective analysis and small number of cases. There was no control group for cases with gastroschisis since all pregnant women were delivered before 35 weeks of gestation. A prediction of simple versus complex gastroschisis is possible using the IABD, but the performance of this model is low. A prospective, randomized, multicenter study could analyze the performance of the measurements in a larger cohort.

To conclude, IABD gives the best prediction for simple versus complex gastroschisis with a cut-off value of 18 mm. EABD cut-off values of 10 mm and 18 mm showed low sensitivity and specificity to predict complex gastroschisis.

## Figures and Tables

**Table 1:** Maternal characteristics and outcomes of cases with gastroschisis.

Parameter		Gastroschisis (*N* = 34)
Age (years)	Mean	23 (17–37)
First examination (weeks of pregnancy)	Mean	22 + 3 (11+4–31+0)
Karyotyping	Not performed	22 (64.7%)
	Performed	12 (35.3%)
	Normal	34
	Abnormal	0
Other anomalies		2 (6.1%)
Outcome	Alive	33
	Intrauterine fetal death	0
	Abortion	0
	Termination	0
	Lost	1
Delivery (weeks of pregnancy)	Mean	33 + 6 (33+0–34+5)
Mode of delivery	Vaginal	0
	C-section	33 (100%)
Weight of the newborn	Mean	2190 (1370–2985)
Weight in percentile	Mean	40 (20–90)

**Table 2:** Prediction of complex gastroschisis with intraabdominal bowel dilatation (IABD) and extra-abdominal bowel dilatation (EABD).

Cut-Off Value	Simple Gastroschisis (*N* = 22)	Complex Gastroschisis (*N* = 11)	Sensitivity %	Specificity %	PPV %	NPV %
IABD < 10 mm	20	8	27.3	90.9	60	71.4
IABD ≥ 10 mm	2	3				
IABD < 18 mm	22	8	27.3	100	100	73.3
IABD ≥ 18 mm	0	3				
EABD < 10 mm	7	3	72.7	31.8	34.8	70.0
EABD ≥ 10 mm	15	8				
EABD < 18 mm	16	6	45.5	72.7	50	84.2
EABD ≥ 18 mm	6	5				
IABD + EABD < 10 mm	7	3	50	77.7	60	70
IABD + EABD ≥ 10 mm	2	3				
IABD + EABD < 18 mm	16	6	33.3	100	100	72.7
IABD + EABD ≥ 18 mm	0	3				

PPV: positive predictive value; NPV: negative predictive value.
